# Microtemporal Dynamics of Dietary Intake, Physical Activity, and Impulsivity in Adult Attention-Deficit/Hyperactivity Disorder: Ecological Momentary Assessment Study Within Nutritional Psychiatry

**DOI:** 10.2196/46550

**Published:** 2023-08-17

**Authors:** Alea Ruf, Andreas B Neubauer, Elena D Koch, Ulrich Ebner-Priemer, Andreas Reif, Silke Matura

**Affiliations:** 1 Department of Psychiatry, Psychosomatic Medicine and Psychotherapy University Hospital Goethe University Frankfurt Germany; 2 DIPF | Leibniz Institute for Research and Information in Education Frankfurt Germany; 3 Center for Research on Individual Development and Adaptive Education of Children at Risk (IDeA) Frankfurt Germany; 4 Mental mHealth Lab Institute of Sports and Sports Science Karlsruhe Institute of Technology (KIT) Karlsruhe Germany; 5 Department of Psychiatry and Psychotherapy Central Institute of Mental Health, Medical Faculty Mannheim Heidelberg University Mannheim Germany

**Keywords:** impulsivity, nutrition, macronutrient intake, physical activity, ecological momentary assessment, EMA, attention-deficit/hyperactivity disorder, ADHD, diet, neurodevelopmental, hyperactivity, macronutrient, psychiatry, symptoms, mobile, impulsivity, mobile phone

## Abstract

**Background:**

Increasing attention is being paid to lifestyle factors, such as nutrition and physical activity (PA), as potential complementary treatment options in attention-deficit/hyperactivity disorder (ADHD). Previous research indicates that sugar and saturated fat intake may be linked to increased impulsivity, a core symptom of ADHD, whereas protein intake and PA may be related to reduced impulsivity. However, most studies rely on cross-sectional data that lack microtemporal resolution and ecological validity, wherefore questions of microtemporal dynamics (eg, is the consumption of foods high in sugar associated with increased impulsivity within minutes or hours?) remain largely unanswered. Ecological momentary assessment (EMA) has the potential to bridge this gap.

**Objective:**

This study is the first to apply EMA to assess microtemporal associations among macronutrient intake, PA, and state impulsivity in the daily life of adults with and without ADHD.

**Methods:**

Over a 3-day period, participants reported state impulsivity 8 times per day (signal-contingent), recorded food and drink intake (event-contingent), and wore an accelerometer. Multilevel 2-part models were used to study the association among macronutrient intake, PA, and the probability to be impulsive as well as the intensity of impulsivity (ADHD: n=36; control: n=137).

**Results:**

No association between macronutrient intake and state impulsivity was found. PA was not related to the intensity of impulsivity but to a higher probability to be impulsive (ADHD: β=−.09, 95% CI −0.14 to −0.04; control: β=−.03, 95% CI −0.05 to −0.01). No evidence was found that the combined intake of saturated fat and sugar amplified the increase in state impulsivity and that PA alleviated the positive association between sugar or fat intake and state impulsivity.

**Conclusions:**

Important methodological considerations are discussed that can contribute to the optimization of future EMA protocols. EMA research in the emerging field of nutritional psychiatry is still in its infancy; however, EMA is a highly promising and innovative approach as it offers insights into the microtemporal dynamics of psychiatric symptomology, dietary intake, and PA in daily life.

## Introduction

### Background

Attention-deficit/hyperactivity disorder (ADHD), initially conceptualized as a neurodevelopmental disorder restricted to childhood, is now recognized as a condition persisting into adulthood, with approximately 2.5% to 3% of adults showing clinically relevant symptoms [1,2]. ADHD is characterized by impaired symptoms of hyperactivity, inattention, and impulsivity. Individuals with ADHD are at risk for poor academic performance [3], accidents [4], financial problems [5], and numerous adverse outcomes (the review by Kooij et al [6] provides an overview). A multimodal and multidisciplinary approach, including pharmacotherapy, cognitive behavioral therapy, psychoeducation, and coaching, should be applied for the treatment of adult ADHD [6]. However, adult ADHD is underdiagnosed and undertreated [6], likely because of the unavailability of diagnostic services or their limited availability in very few specialized facilities [7]. Despite the high efficacy of pharmacotherapy for the short-term treatment of ADHD [8], not every patient responds to medication (eg, 24% of nonresponders [9]), long-term effects of pharmacotherapy are understudied [8], and some studies suggest that pharmacological treatment is positively associated with symptom severity in the long term [10]. Furthermore, pharmacotherapy is associated with several side effects such as increased heart rate and blood pressure [11], reduced appetite [12], and sleep problems (for an overview, refer to the review by Stein et al [13]), and adherence to pharmacological treatment is often low [14]. These disadvantages and challenges of pharmacotherapy highlight the need for easily accessible complementary treatment options for adults with ADHD. Lifestyle factors, such as nutrition and physical activity (PA), might be promising targets for the development of complementary treatments [15-17].

### Nutrition, Impulsivity, and ADHD

Although it is well known that nutrition has a significant impact on physical health, evidence is growing that nutrition also plays an important role in mental health and mental functioning, wherefore the emerging field of nutritional psychiatry is attracting growing attention [18]. For instance, the Mediterranean diet seems to have protective effects against depressive symptoms [19] and may even be an effective treatment strategy for depression [20]. Increasing attention is also being paid to the role of nutrition in ADHD (for an overview, refer to the reviews by Breda et al [15] and Pinto et al [17]). Del-Ponte et al [21] conducted a meta-analysis and found that healthy dietary patterns were associated with a decreased risk for ADHD (odds ratio [OR] 0.65, 95% CI 0.44-0.97), whereas unhealthy dietary patterns were associated with an increased risk for ADHD (OR 1.41, 95% CI 1.15-1.74). The authors concluded that the findings suggest that healthy diets rich in fruits and vegetables can protect against ADHD, whereas diets high in refined sugar and saturated fat can increase the risk [21]. These findings were confirmed by a recent meta-analysis that found that healthy dietary patterns characterized by fruits, vegetables, and fish were associated with a reduced risk for ADHD (OR 0.63, 95% CI 0.41-0.96), whereas Western dietary patterns, consisting of red meat, processed meat, animal fat, and salt (OR 1.92, 95% CI 1.13-3.26), and junk food dietary patterns, including sweets, sweetened beverages, snacks, ice creams, and fast foods (OR 1.51, 95% CI 1.06-2.16), were found to be associated with an increased risk for ADHD [22]. A positive association between total sugar intake (ie, from sugar-sweetened beverages and dietary sources) and the risk of ADHD was found in another meta-analysis [23]. Although these meta-analytical findings support the presence of a link between nutrition and ADHD, most of the included studies do not allow causal conclusions. Cross-sectional studies do not provide information on the directionality of the association and lack microtemporal resolution and ecological validity. More high-quality studies (eg, randomized and microrandomized controlled trials) are needed to gain insights into causality and underlying mechanisms. As ADHD symptomology is dynamic in nature [24], studies of high temporal resolution and ecological validity, such as ecological momentary assessment (EMA) studies, are required to obtain evidence on short-term, microtemporal associations in daily life. EMA comprises repeated assessments of behaviors (eg, food intake), experiences (eg, impulsivity), and physiological parameters throughout a day in the moment and in the natural environment, which enables studying complex psychological, behavioral, and physiological processes [25].

Evidence indicates that nutrition is not only associated with ADHD per se but also with core symptoms of ADHD, such as impulsivity and executive dysfunction. Impulsivity is a multidimensional construct [26] that manifests as “impatience, acting without thinking, spending impulsively, starting new jobs and relationships on impulse, and sensation seeking behaviours” [27]. Impulsivity is closely linked to impaired executive function [28]. Accordingly, the concepts of impulsivity [28] and executive dysfunction [29] have been used to describe the same impairments in ADHD. A cross-sectional study among a nonclinical adult sample found an association between fast food consumption and greater impulsivity (ie, delay discounting, a behavioral measure of impulsivity) [30]. However, directionality remains unclear. Further studies in nonclinical samples of young adults found an association between a Western-style diet high in saturated fat and added sugar and greater trait impulsivity, with hypothesized bidirectional causation [31], and a relationship between stronger inhibitory control and lower consumption of foods high in saturated fat [32]. Research in rodents provides the first evidence on the causality of the relationship between nutrition and impulsivity by showing that a high-fat and high-sugar diet increases impulsivity [33,34].

In contrast to fat and sugar intake, protein intake appears to have beneficial effects on executive function. A randomized controlled dietary intervention trial in healthy men found improved reaction times on the “Go/No-Go task,” a measure of response inhibition and a proxy for impulsivity, in the group that followed a high-protein diet for 3 weeks [35]. Tryptophan, an essential amino acid and precursor to serotonin found in almost all proteins, has been discussed as a potential underlying mechanism. For instance, it was found that dietary intake of tryptophan was associated with lower levels of one facet of emotion-related impulsivity (ie, Pervasive Influence of Feelings) [36].

Although many studies have assessed the relationship between ADHD symptomology or impulsivity and long-term dietary exposure (eg, habitual food intake captured through food frequency questionnaires [FFQs]), there is also some evidence that the intake of certain macronutrients has short-term effects on executive function. For instance, Brandley and Holton [37] showed that a nutritionally balanced breakfast with an optimal macronutrients ratio (ie, 25% fat, 45% carbohydrate, and 29% protein) improved executive function of college students with and without ADHD an hour after consumption.

### PA, Impulsivity, and ADHD

Not only nutrition but also PA seems to influence impulsivity and executive function as PA is associated with reduced impulsivity. A meta-analysis found a significant overall effect of acute, but not chronic physical exercise on executive functions in children, adolescents, and young adults [38]. Several meta-analyses have studied the effect of exercise on cognition and behavior in children with ADHD showing that exercise has positive effects on executive functions [39-41]. Research assessing the effect of PA in adults with ADHD remains limited [39,42]. However, the initial findings are promising. For instance, a cross-sectional pilot study found that adults with ADHD who engaged in frequent aerobic PA reported significantly lower levels of behavioral impulsivity [43]. In addition, the first evidence indicates that noncardio PA (ie, whole body vibration) has positive effects on cognitive functioning in adults with ADHD [44,45]. A study in college students with and without ADHD showed that in those with ADHD inhibitory performance, one aspect of executive function improved after acute exercise; however, all aspects of executive functions improved in those without ADHD [46]. In a study following a counterbalanced repeated measures design with a control condition, adults with ADHD improved reaction times in congruent and incongruent trials of the flanker task after 30 minutes of continuous stationary cycling, indicating benefits of acute exercise [47].

### Objectives

ADHD is characterized by dynamic symptoms that manifest as states of hyperactivity, inattention, and impulsivity [24]. However, most studies rely on cross-sectional data that lack microtemporal resolution and do not provide evidence with regard to true temporal associations. Retrospective reports (eg, FFQs and trait questionnaires) are prone to cognitive biases—potentially, in particular, in individuals with ADHD—and overlook fluctuations of ADHD symptoms [48]. It remains unstudied whether the intake of certain macronutrients or the engagement in PA alters impulsivity within minutes or hours in everyday life. EMA can bridge this gap, as it offers great potential to provide novel insights into ADHD symptomatology in daily life to better understand behavior and functioning at the intraindividual level [24,48]. Despite the need for studies of high temporal resolution and ecological validity and the potential of EMA, so far, no study has applied EMA to assess whether the intake of certain macronutrients and engagement in PA are associated with changes in state impulsivity in daily life. Therefore, the aim of this study was to apply EMA to assess short-term, microtemporal associations between macronutrient intake, PA, and state impulsivity in daily life of adults with and without ADHD. On the basis of previous research, six research questions were elaborated. We examined whether (1) the intake of sugar and (2) the consumption of saturated fats are associated with an increase in state impulsivity, and whether (3) the intake of proteins and (4) the engagement in PA are associated with decreased state impulsivity. As there is evidence that the combination of a high-fat and high-sugar diet was associated with ADHD and impulsivity [21,31,33], we assessed whether (5) the combined intake (ie, the interaction) of saturated fat and sugar amplifies the increase in state impulsivity. Intriguingly, research indicates that PA may buffer against the adverse effects of fat intake on cognitive functioning [49,50]. Therefore, we investigated whether (6) PA alleviates the positive association between sugar or fat intake and state impulsivity.

## Methods

### Procedure

Data were collected within the Eat2beNICE-APPetite study, which comprises 2 in-person sessions as well as an EMA period (parts of the data of this study have been used for different research questions [51-53]). In the first in-person session, participants completed questionnaires and received comprehensive training to familiarize them with the APPetite mobile app, which was used for the EMA period (refer to the study by Ruf et al [51] for further details). Body weight and height were measured to calculate BMI.

### Ethics Approval

The local ethics committee of the faculty of medicine of the Goethe University Frankfurt (Ethikkommission des Fachbereichs Medizin der Goethe-Universität) approved the study (reference number: 192/18).

### Informed Consent

All participants declared that they understood the study procedure and signed a written informed consent. This study was conducted in accordance with the Code of Ethics of the World Medical Association (Declaration of Helsinki, 1975).

### EMA Protocol

The participants used a study smartphone to complete the EMA protocol of the APPetite mobile app for 3 consecutive days (2 weekdays and 1 weekend day). Participants received 8 semirandom signal-contingent prompts per day (between 8 AM and 10 PM, with at least 1 hour in-between prompts). Each prompt assessed state impulsivity. The prompts could be postponed for up to 25 minutes. Participants were able to record food intake at any time (ie, event-contingent) through the incorporated APPetite-food record. In addition, a time-contingent prompt at 9 PM asked participants whether all foods and drinks consumed during the day were recorded. Further details on the APPetite mobile app can be found in the study by Ruf et al [51].

### Sample

The Eat2beNICE-APPetite study recruited participants from 4 existing study cohorts. Adults with ADHD were invited from (1) the PROUD (Prevention of Comorbid Depression and Obesity in Attention-Deficit/Hyperactivity Disorder) study [54]; (2) the BipoLife-A1 study that follows up individuals with an increased risk for bipolar disorders, including patients affected by ADHD or depression (or both) [55,56]; and (3) the PROBIA (Treating Impulsivity with Probiotics in Adults) study, which recruited patients with ADHD or borderline personality disorder (or both) [57]. Healthy controls were recruited from the Longitudinal Resilience Assessment study, which enrolled individuals not affected by psychiatric conditions and followed them up since 2016 [58].

In total, 43 adults with ADHD and 185 adults without ADHD participated in the study. After the first in-person session, 4 participants without ADHD dropped out because of personal reasons (eg, spontaneous vacation) or the inability to respond to prompts (eg, because of work commitments). Data of 1 participant without ADHD were excluded as they proved to be untrue. Data of 26 participants without ADHD and 6 participants with ADHD were excluded because of incomplete records of food intake (eg, only 1 meal recorded). One participant in the ADHD sample had to be excluded because no PA data could be retrieved because of technical problems. One participant without ADHD was excluded because BMI was unavailable owing to scale malfunction. Furthermore, 16 participants without ADHD were excluded from the analyses because they showed no variation in state impulsivity across prompts (ie, all Momentary Impulsivity Scale [MIS] items consistently answered with *1—not applicable*). The final sample included 36 participants with ADHD and 137 participants without ADHD. Demographics of the ADHD and control groups are presented in Table 1. The samples differed in terms of gender (χ*^2^*_1_=4.5, *P*=.03), age (Mann-Whitney *U*=1667; *P*=.002), and BMI (kg/m^2^; Mann-Whitney *U*=1433; *P*<.001).

**Table 1:** Demographics of the sample with and without attention-deficit/hyperactivity disorder (ADHD).

			ADHD sample (n=36)		Control sample (n=137)
**Gender, n (%)**					
	Woman	19 (53)		100 (73)	
	Man	17 (47)		37 (27)	
Age (years), mean (SD)			35.25 (12.04)		28.8 (7.72)
BMI (kg/m^2^), mean (SD)			29.06 (7.87)		24.08 (4.14)

### Measures

#### Macronutrient Intake

Macronutrient intake was captured using the APPetite mobile app, which comprises a food record [51]. The food recording follows a six-step process: (1) selection of meal type, (2) entry of time of intake, (3) selection of consumed foods and drinks, (4) specification of consumed amounts, (5) presentation of reminders for commonly forgotten foods, and (6) indication of the predominant reason for eating or drinking. Participants were instructed to record their food and drink as soon as possible after consuming them. To generate nutritional values (ie, sugar, saturated fat, and protein intake), the collected food entries were transferred to myfood24-Germany [59] by trained staff. A feasibility, usability, and validation study was conducted to evaluate the APPetite mobile app. The findings indicated that the APPetite mobile app is a feasible and valid dietary assessment tool that is more accurate compared with 24-hour recalls [51].

#### State Impulsivity

State impulsivity was assessed using the MIS [60]. The MIS captures state impulsivity on the basis of 4 items, each of which comprises a statement (eg, “I said things without thinking”). Participants rated how well each statement described their behavior, cognition, and experiences since the last prompt or since waking up in the first daily prompt on a 5-point scale. A sum score of the items was calculated. Higher values indicate greater state impulsivity. Note that the original response scale (1=very slightly or not at all, 2=a little, 3=moderately, 4=quite a bit, and 5=extremely) was slightly altered during translation (translate–back-translate procedure with a native bilingual speaker in English and German) because the literal translation lacked differentiability. One main difference between the English and German version is that *1* on the response scale stands for *not applicable* in the German version (1=nicht zutreffend ["not applicable" in German], 2=eher nicht zutreffend ["partially not applicable" in German], 3=teils-teils ["half and half" in German], 4=eher zutreffend ["partially applicable" in German], and 5=zutreffend ["applicable" in German]). In the final data sets, the McDonald ω of the MIS was 0.576 (within) and 0.832 (between) in the ADHD sample and 0.505 (within) and 0.768 (between) in the control sample.

#### Trait Impulsivity

The UPPS-P Impulsive Behavior Scale [61,62] was used to assess trait impulsivity based on 59 items. Each item described a statement (eg, “I have trouble controlling my impulses”). Participants reported how well each statement described them on a 4-point scale from *agree strongly* to *disagree strongly*. The UPPS-P Impulsive Behavior Scale assesses impulsivity as a multifaceted construct that includes the following subscales: negative urgency (12 items), positive urgency (14 items), lack of premeditation (11 items), lack of perseverance (10 items), and sensation seeking (12 items). The German translation of the items for the scales negative urgency, lack of premeditation, lack of perseverance, and sensation seeking were taken from Schmidt et al [63]. The items of the subscale positive urgency were translated through the translate-back-translate procedure. In this study’s samples, internal consistency was ADHD, α=.92 and control, α=.89 for negative urgency; ADHD, α=.81 and control, α=.75 for premeditation; ADHD, α=.83 and control, α=.83 for perseverance; ADHD, α=.89 and control, α=.85 for sensation seeking; and ADHD, α=.93 and control, α=.92 for positive urgency.

#### Physical Activity

PA was captured objectively using Move 3 sensors (movisens GmbH). Participants wore the sensor on their nondominant wrist during the EMA period (day and night). The software DataAnalyzer (version 1.13.7; movisens GmbH) was used to calculate the movement acceleration intensity per minute (mg/min) from raw accelerometry. Nonwear time was excluded from the analysis.

### Data Preprocessing

A total of 13 single days of the control sample had to be excluded owing to incomplete dietary data. Data preprocessing was completed based on the time intervals for which state impulsivity was assessed (ie, time between current prompt and previous prompt or waking up). To study the association between macronutrient intake, PA, and state impulsivity, each of these time interval was matched to concurrent sugar, saturated fat, and protein intake and mean PA (ie, mean movement acceleration). Concurrent intake was defined as the sum of any intake of sugar, saturated fat, or protein within the respective time interval. On the basis of the movement acceleration intensity per minute (mg/min), the mean movement acceleration was calculated for each time interval in which the sensor was worn for at least two-thirds of the time.

The level-1 predictors sugar, fat, and protein intake and PA were person mean centered to produce unbiased estimates of the within-person effect [64]. To avoid estimation problems owing to substantial differences in the variance of the predictors and the outcome, the level-1 predictors were divided by 10. The level-2 covariates age and BMI were centered at 30 and 25, respectively, to make the model intercept more interpretable as recommended by Viechtbauer [65]. Grand mean centering was used for level-2 covariate trait impulsivity. The level-2 covariate gender was coded as 0 (male) and 1 (female).

The MIS items were not completed at 401 time intervals (control: n=297; ADHD: n=104) and were therefore excluded. Owing to the semirandom sampling protocol, the time intervals varied in length. Beyond that, the option to postpone prompts and the assessment of state impulsivity “since waking up” in the first prompt produced rather short or long time intervals. As we did not expect an effect of macronutrient intake and PA on state impulsivity within <15 minutes [66] and were interested in short-term associations, time intervals shorter than 15 minutes (control: n=21; ADHD: n=8) and longer than 3 hours (control: n=118; ADHD: n=27) were excluded. In addition, time intervals in which the level-1 predictor PA was not available (eg, because of the exclusion criteria for time intervals in which the sensor was worn <two-thirds of the time), were excluded (control: n=166; ADHD: n=73). The final data set included 629 time intervals in the ADHD and 2464 in the control sample and is provided in Multimedia Appendix 1.

### Data Analysis

Owing to the nested data structure (time intervals [level 1] nested within individuals [level 2]), multilevel models were needed for the analyses. The MIS score, the outcome of this study, showed a strongly right-skewed distribution that did not meet the assumptions of linear multilevel modeling. Furthermore, owing to a significant proportion of the lowest MIS score (all items answered with 1 *not applicable*, resulting in an MIS score of 4), a gamma multilevel model was unable to represent the right skew in the data adequately. To account for the inflation of the lowest MIS score (ie, 4), which represents the absence of impulsivity, we used a multilevel 2-part model that allows to account for zero-inflated, continuous data (ie, semicontinuous data). This type of model allows studying whether the intake of certain macronutrients and PA is associated with the occurrence of state impulsivity (ie, is an individual impulsive at all?) and the intensity of impulsivity (ie, if an individual is impulsive, how impulsive are they?). To move the inflation from 4 to 0, the 5-point scale of the MIS was recoded (1 to 0, 2 to 1, 3 to 3, 4 to 3, and 5 to 4). The model we applied combines a multilevel logistic regression in the zero part to study the occurrence of state impulsivity and a multilevel gamma regression (to account for the right skew in the positive values) in the continuous part of the model to assess the intensity of state impulsivity. The model does not only allow to study the occurrence and intensity of state impulsivity but also accounts for the potential dependency between the 2 outcome components by modeling a cross-part correlation. Although logistic regressions typically predict the outcome to be 1, the multilevel logistic regression in the zero part of the model used in this study predicts no impulsivity (outcome=0), that is, the probability not to be impulsive in a given individual in a given time interval.

To examine the associations between macronutrient intake, PA, and state impulsivity (research question 1 to 4), a model with the level-1 predictors sugar, saturated fat, and protein intake and PA in both model parts (ie, the logistic regression as well as the gamma regression) was run. A joint model was chosen to control for the other predictors, as the intake of different macronutrients and PA naturally does not occur in isolation, but in combination. Next, the interaction between the level-1 predictors sugar and fat intake was added to both model parts (research question 5). Finally, a model, including the interaction between sugar intake, fat intake, and PA (ie, 4 interactions modeled: 2-way interactions between sugar intake and PA, between fat intake and PA, between sugar and fat intake, and 3-way interaction among sugar intake, fat intake, and PA) in both model parts was run (research question 6). In all models, the level-2 covariates gender, age, BMI, and trait impulsivity were included. All models included random intercepts in both model parts (ie, we expect individuals to differ in their average probability not to be impulsive and the average intensity of state impulsivity) and random slopes for all level-1 predictors (and their interaction) to examine whether the effects differ between individuals. The 3 models were run separately for the ADHD and the control group.

All models were estimated using the R-package *brms* [67,68], which supports Bayesian multilevel modeling. Credible intervals (95% CI) of fixed effects that do not include 0 were interpreted as significant effects. As nonpositive estimates for SDs are not allowed, the lower limit of the CI of random effects that are equal to 0 suggest that the random effect is not significant (ie, that individual differences in the intercept, the effects of the level-1 predictors, or the interactions between level-1 predictors are small and possibly not statistically meaningful). Details on the model used in this study (eg, implementation and interpretation) can be found in the study by Ruf et al [52].

The estimation of model parameters was based on 10,000 iterations. The initial values for the sampler were set to 0 (*init=0*) and the maximum tree depth was set to 11 to reach convergence in 2 models (see the open R code provided in Multimedia Appendix 2). The default settings of all other sampling and prior parameters were maintained. R (version 4.2.2; R Foundation for Statistical Computing [69]), RStudio (version 2022.7.2.576; Posit [70]), *brms* (version 2.18.0), and *rstan* (version 2.26.13) [71] were used to perform the analyses.

## Results

### Descriptive Findings

Descriptive statistics of the level-1 predictors sugar, saturated fat, and protein intake and PA and the level-2 covariate trait impulsivity for the ADHD sample and the control sample are shown in Table 2. Participants reported not to be impulsive (MIS=0) in 28.6% (180/629) of the time intervals in the ADHD sample and in 58.2% (1434/2464) of the time intervals in the control sample. Within the time intervals in which participants reported to be impulsive (ADHD: n=449; control: n=1030), state impulsivity was rated on average 3.9 (SD 2.6) in the ADHD sample and 2.8 (SD 1.9) in the control sample on the shifted response scale (ranging from 0 to 16). To test whether individuals with and without ADHD differed in the occurrence and the intensity of impulsivity, a multilevel 2-part model with the level-2 predictor ADHD diagnosis (0=no ADHD, 1=ADHD) including all participants was calculated (see model 0 in the open R code provided in Multimedia Appendix 2). Results showed that individuals with ADHD were less likely not to be impulsive (ie, significant fixed effect of ADHD diagnosis in the zero part: −1.69, SE 0.32, 95% CI −2.34 to −1.06) and reported significantly higher levels of impulsivity intensity (ie, significant fixed effect of ADHD diagnosis in the continuous part: 0.36, SE 0.08, 95% CI 0.20-0.51) compared with individuals without ADHD.

Average compliance with the signal-contingent prompts (ie, percentage of complete prompts within received prompts) was 89.6 (SD 12.4) in the ADHD and 90.3 (SD 11.5) in the control sample (not including participants and days that were excluded as a whole, but including time intervals that were excluded from the final analyses based on interval length and missing PA or MIS).

**Table 2:** Descriptive statistics of the level-1 predictors (control: n=2464; ADHD: n=629) and level-2 covariate trait impulsivity (control: n=137; ADHD: n=36).

		ADHD^a^ sample		Control sample	
		Values, mean (SD)	Values, median^b^ (range)	Values, mean (SD)	Values, median (range)
**Level 1**					
	Sugar intake in g	10.19 (17.50 [overall]; 5.71 [between])	0 (0-101.05)	9.87 (15.94 [overall]; 4.41 [between])	1.04 (0-186.67)
	Saturated fat intake in g	4.46 (8.97 [overall]; 2.39 [between])	0 (0-76.15)	4.47 (7.92 [overall]; 2.11 [between])	0.03 (0-65.23)
	Protein intake in g	10.42 (20.34 [overall]; 4.95 [between])	0 (0-145.2)	9.93 (16.73 [overall]; 4.13 [between])	0.64 (0-137.09)
	PA^c^ (acceleration) in mg	130.85 (77.07 [overall]; 38.04 [between])	120.63 (8.64-815.3)	144.68 (78.77 [overall]; 31.68 [between])	134.13 (10.23-1445.94)
**Level 2**					
	Trait impulsivity	30.24 (5.08)	30.1 (18.2-40.0)	23.78 (3.68)	23.4 (16.4-35.8)

^a^ADHD: attention-deficit/hyperactivity disorder.

^b^Median values were included to highlight that the level-1 predictors followed skewed distributions and were zero inflated (ie, food intake did not occur within each time interval, where macronutrient intake is equal to 0).

^c^PA: physical activity.

### Findings From the Multilevel 2-Part Models

#### Interpretation Overview

As the estimates of the continuous part (ie, the gamma regression) of the multilevel 2-part model are modeled on the log scale, the exponential is used to obtain estimates in the original metric. In the zero part (ie, the logistic regression), estimates were modeled on the logit scale. The intercept of the zero part represents the average log-odds of no impulsivity across all participants when all the predictors were 0. The inverse logit function (eg, the *plogis* function in R) can be used to transform the log-odds to the probability not to be impulsive. The predictor estimates in the zero part represent the expected change in the log-odds of no impulsivity for a 1-unit increase in each predictor, respectively. To obtain the expected change in the probability not to be impulsive, the probability of the intercept (ie, *plogis*[intercept]) can be compared with the predicted probability when the respective predictor takes on a certain value (eg, if the chosen value of the predictor is 1, the predicted probability is *plogis*[intercept + fixed effect of the predictor]).

#### Sugar, Saturated Fat, and Protein Intake; PA; and State Impulsivity

Results of the model, including fixed and random effects for sugar, saturated fat, and protein intake and PA in both model parts are shown in Table 3 for the ADHD sample. The intercept of the zero part indicates that the mean probability not to be impulsive is 11.7% (*plogis*[−2.02]) when all predictors and covariates are equal to 0. Sugar, saturated fat, and protein intake had no significant fixed effect on the probability not to be impulsive. However, the effect of saturated fat and protein intake on the probability of no impulsivity differs across individuals with an *SD* of 0.29 and 0.22, respectively. In time intervals in which PA is 1 unit (ie, 10 mg) above 0 (ie, above the person mean) and all other predictors are 0, the probability not to be impulsive is 10.8% (*plogis*[−2.02−0.09]), that is, a 1-unit increase in PA is associated with a decrease in the probability not to be impulsive of 0.9% (11.7%−10.8%=0.9%) when all other predictors are 0. This indicates that higher levels of PA are associated with a higher probability to be impulsive. The intercept of the continuous part of the model demonstrates that when all predictors and covariates are equal to 0, participants with ADHD report an average impulsivity intensity of 3.32 (e^1.20^). There was no significant fixed effect of sugar, saturated fat, and protein intake and PA. Accordingly, the intake of sugar, saturated fat, and protein intake and the level of PA were not associated with the intensity of state impulsivity. There was a negative cross-part correlation (−0.38, SE 0.17, 95% CI −0.68 to −0.03) indicating that individuals who are impulsive more often are more impulsive when they are impulsive suggesting that the frequency and intensity of impulsivity correlate.

**Table 3:** Model estimates of the multilevel 2-part model including fixed and random effects for sugar, saturated fat, and protein intake as well as physical activity (PA) in both model parts in the attention-deficit/hyperactivity disorder sample.

Model 1			Zero part, estimate (SE; 95% CI)		Continuous part, estimate (SE; 95% CI)
**Fixed effects**					
	Intercept	−2.02 (0.55; −3.14 to −0.97)		1.20 (0.12; 0.96 to 1.44)	
	Sugar intake	−0.01 (0.10; −0.22 to 0.18)		0.01 (0.02; −0.03 to 0.04)	
	Saturated fat intake	−0.10 (0.29; −0.70 to 0.45)		0.01 (0.04; −0.07 to 0.10)	
	Protein intake	−0.09 (0.14; −0.38 to 0.16)		−0.02 (0.02; −0.05 to 0.01)	
	PA	−0.09 (0.03; −0.14 to −0.04)		0.00 (0.00; −0.00 to 0.01)	
	Gender	0.40 (0.74; −1.04 to 1.89)		−0.15 (0.17; −0.48 to 0.20)	
	Age (years)	−0.02 (0.04; −0.09 to 0.05)		0.00 (0.01; −0.01 to 0.02)	
	BMI (kg/m^2^)	0.11 (0.05; 0.01 to 0.20)		0.00 (0.01; −0.02 to 0.02)	
	Trait impulsivity	−0.11 (0.07; −0.24 to 0.03)		0.03 (0.02; 0.00 to 0.07)	
**Random effects**					
	SD (intercept)	1.83 (0.32; 1.29 to 2.55)		0.44 (0.06; 0.33 to 0.58)	
	SD (sugar intake)	0.12 (0.09; 0.00 to 0.34)		0.03 (0.02; 0.00 to 0.07)	
	SD (saturated fat intake)	0.29 (0.24; 0.01 to 0.89)		0.04 (0.03; 0.00 to 0.11)	
	SD (protein intake)	0.22 (0.14; 0.01 to 0.55)		0.02 (0.01; 0.00 to 0.04)	
	SD (PA)	0.06 (0.04; 0.00 to 0.14)		0.01 (0.01; 0.00 to 0.03)	

Table 4 shows the results of the same model (ie, fixed and random effects for sugar, saturated fat, and protein intake and PA in both model parts) for the control sample. Individuals without ADHD had a mean probability not to be impulsive of 69.2% (*plogis*[0.81]) when all predictors and covariates were equal to 0. Similar to the ADHD sample, sugar, saturated fat, and protein intake had no significant fixed effect on the probability not to be impulsive. However, in contrast to the ADHD sample, the effects of saturated fat and protein intake on the probability not to be impulsive did not differ among individuals without ADHD. However, higher levels of PA were also associated with a higher probability to be impulsive in adults without ADHD. Accordingly, the probability not to be impulsive is 68.6% (*plogis*[0.81−0.03]) in time intervals in which PA is 1 unit (ie, 10 mg) above 0 (ie, above the person mean) and all other predictors are 0. Consequently, a 1-unit increase in PA is associated with a decrease in the probability not to be impulsive by 0.6% (69.2%−68.6%=0.6%) when all other predictors in the model are 0. Again, the cross-part correlation (−0.53, SE 0.09, 95% CI −0.69 to −0.33) was negative, indicating that individuals who were impulsive more often were more impulsive when they were impulsive.

**Table 4:** Model estimates of the multilevel 2-part model, including fixed and random effects for sugar, saturated fat, and protein intake as well as physical activity (PA) in both model parts in the control sample.

Model 2		Zero part, estimate (SE; 95% CI)	Continuous part, estimate (SE; 95% CI)
**Fixed effects**			
	Intercept	0.81 (0.27; 0.28 to 1.35)	0.68 (0.07; 0.54 to 0.83)
	Sugar intake	−0.02 (0.04; −0.10 to 0.06)	−0.01 (0.01; −0.04 to 0.02)
	Saturated fat intake	−0.04 (0.11; −0.25 to 0.18)	0.00 (0.04; −0.07 to 0.08)
	Protein intake	−0.04 (0.05; −0.13 to 0.06)	−0.02 (0.02; −0.05 to 0.02)
	PA	−0.03 (0.01; −0.05 to −0.01)	0.01 (0.00; 0.00 to 0.02)
	Gender	−0.52 (0.33; −1.17 to 0.12)	0.17 (0.09; −0.00 to 0.34)
	Age (years)	−0.02 (0.02; −0.05 to 0.02)	−0.00 (0.00; −0.01 to 0.01)
	BMI (kg/m^2^)	0.03 (0.04; −0.04 to 0.11)	0.00 (0.01; −0.02 to 0.02)
	Trait impulsivity	−0.20 (0.04; −0.27 to −0.13)	0.05 (0.01; 0.03 to 0.07)
**Random effects**			
	SD (intercept)	1.44 (0.12; 1.22 to 1.69)	0.33 (0.03; 0.27 to 0.39)
	SD (sugar intake)	0.06 (0.04; 0.00 to 0.17)	0.03 (0.02; 0.00 to 0.06)
	SD (saturated fat intake)	0.14 (0.10; 0.00 to 0.38)	0.05 (0.04; 0.00 to 0.14)
	SD (protein intake)	0.05 (0.04; 0.00 to 0.15)	0.02 (0.02; 0.00 to 0.06)
	SD (PA)	0.07 (0.02; 0.04 to 0.10)	0.01 (0.01; 0.00 to 0.02)

#### Interaction Between Sugar and Saturated Fat Intake

To study whether the combined intake of saturated fat and sugar amplifies the effect of sugar and saturated fat intake on state impulsivity, the interaction between the level-1 predictors sugar and fat intake was added to both models. Results of the ADHD sample are shown in Table S1 in Multimedia Appendix 3 and results of the control sample are shown in Table S2 in Multimedia Appendix 3. In both samples and both model parts, the interaction between sugar and saturated fat intake was not significant. However, in the ADHD sample, the interaction effect between sugar and saturated fat intake on the probability not to be impulsive varied across participants with an SD of 0.25 in the zero part.

#### Buffering Effect of PA on the Association Between Sugar and Fat Intake and State Impulsivity

To test whether PA alleviates the positive association between sugar or fat intake and state impulsivity, a model that included the interaction between sugar intake, fat intake, and PA (ie, 4 interactions: 2-way interactions between sugar intake and PA, between fat intake and PA, between sugar and fat intake, and 3-way interaction among sugar intake, fat intake, and PA) in both model parts was run for each sample. Table S3 in Multimedia Appendix 3 shows the results of the ADHD sample and Table S4 in Multimedia Appendix 3 shows the results of the control sample. In both samples, the 4 interactions in the zero and the continuous part of the model were not significant. Only in the ADHD sample, the interaction effect of sugar and saturated fat intake (as in the previous model) and the 3-way interaction among sugar and saturated fat intake and PA varied significantly between participants with ADHD.

## Discussion

### Principal Findings

Although impulsivity, a core symptom of ADHD, can contribute to the disruption of daily functioning, the first evidence indicates that the intake of certain macronutrients and the engagement in PA might alter impulsivity and executive function. However, despite the potential of digital and mobile technologies, studies of high temporal resolution and ecological validity are lacking, and it remains unanswered whether the intake of certain macronutrients and the engagement in PA are associated with short-term changes in state impulsivity in everyday life. Therefore, this study applied EMA to assess short-term, microtemporal dynamics of macronutrient intake, PA, and state impulsivity in daily life of adults with and without ADHD. Contrary to previous findings that suggest that the intake of sugar and saturated fat is associated with greater impulsivity [30-34], whereas the intake of proteins is linked to decreased impulsivity and improved executive function [35,36], no association between macronutrient intake and state impulsivity (ie, the probability to be impulsive and the intensity of impulsivity) was found in this study. However, some between-person variability was observed. Furthermore, in contrast to prior research indicating that PA is associated with reduced impulsivity [38-40], no relationship between PA and the intensity of impulsivity was found and PA was associated with an increased probability to be impulsive in both samples. No evidence was found that the combined intake of saturated fat and sugar amplified the increase in state impulsivity and that PA alleviated the positive association between sugar or fat intake and state impulsivity.

One reason for not finding an association between macronutrient intake and state impulsivity in this study could be the (varying) length of the time intervals in which the association was studied. In this context, several methodological considerations need to be discussed. (1) Assessment of state impulsivity: as 3 items of the MIS describe specific actions (“I said things without thinking,” “I spent more money than I meant to,” “I made a ‘spur of the moment’ decision”), the assessment has to be based on time intervals (here “since the last prompt”). Only the item “I have felt impatient” could be adapted to allow an assessment on the momentary level (ie, “Right now I feel impatient”). A momentary assessment would allow to specify time intervals before the impulsivity assessment more flexibly to study the temporal sequence of the association. (2) Lengths of time intervals: a semirandom signal-contingent EMA protocol was used to assess state impulsivity, so that participants could not predict the exact time of the next prompt. This allowed capturing a better reflection of the participants’ daily lives [72]. However, it results in time intervals of different lengths (with at least 1 hour in-between 2 prompts in this study). However, the first daily assessment of state impulsivity “since waking up” and the postponement of prompts led to even shorter or rather long time intervals. Consequently, the length of time intervals varied considerably. Therefore, time intervals shorter than 15 minutes and longer than 3 hours were excluded from analysis. (3) Temporal manifestation of the effect of macronutrients: research providing evidence regarding the time frame in which macronutrients affect cognition and behavior is still lacking. Beyond that, the time frame in which the effects occur might differ across macronutrients. As the assessment of impulsivity was based on time intervals, this study was restricted to assessing the association between state impulsivity and macronutrient intake within each time interval for which impulsivity was assessed (ie, impulsivity and concurrent macronutrient intake, not intake and subsequent impulsivity). Considering time intervals were of rather different length (15 min to 3 h), the approach of this study might overlook the effect of macronutrient intake. As it is not yet clear within which time frame specific macronutrients affect cognition and behavior, controlled studies are needed to establish the temporal manifestation of the effects of macronutrients on impulsivity. This knowledge is required to adjust EMA protocols to study the relationship between macronutrient, PA, and state impulsivity more systematically. For instance, a time or interval-contingent assessment of state impulsivity could be considered to obtain time intervals of similar length (eg, prompts every hour) [72]. The intervals between prompts should be determined on basis of the novel input and evidence that controlled studies can offer in terms of the temporal manifestation of short-term macronutrients’ effects on impulsivity.

The operationalization of state impulsivity is also important in this context. This study used a self-report measure to capture state impulsivity [60]. However, it remains unclear whether macronutrient intake alters impulsivity, or whether the effect of macronutrients might only affect subdomains (eg, response inhibition). Future studies should include impulsivity-related behavioral measures (eg, stop-signal task, the Digital Marshmallow Test [73], and mobile Balloon Analogue Risk Task [74]), which also open up the opportunity to assess impulsivity momentarily. The momentary assessment of impulsivity, in turn, enables to assess the temporal sequence of the association. In addition, it can contribute to establishing time windows within which macronutrients might affect state impulsivity, as the length of time intervals, within which the association is studied, can be set independently.

Although no overall association between macronutrient intake and state impulsivity was found, some variation (ie, individual differences) in the effect of certain macronutrients was observed, particularly in the ADHD sample. Muth and Park [75] pointed out that age, PA, and glucose metabolism are potential factors contributing to individual differences in the macronutrient-cognition relationship. In this study, PA did not moderate the relationship between sugar and fat intake and state impulsivity. However, future studies should aim at identifying factors that moderate the macronutrient-impulsivity relationship. In this context, continuous glucose monitoring could be a feasible and promising addition to future EMA studies.

In contrast to the expectation, PA was not associated with reduced impulsivity (ie, lower probability to be impulsive and decreased intensity of impulsivity), but with an increase in the probability to be impulsive in adults with and without ADHD. However, it is important to note that temporal associations do not reflect causality. For instance, an individual might experience a state of impulsivity and, consequently, actively decide to engage in PA as a counteracting measure, resulting in a positive association between PA and the probability to be impulsive. Hence, the observed association does not imply that engaging in PA leads to a higher probability to be impulsive. Furthermore, these findings might be a result of the operationalization of PA. Using the mean acceleration within the time intervals might average out relevant fluctuations in PA intensity. Hence, it cannot be differentiated between time intervals in which an individual shows low levels of PA continuously and time intervals in which an individual engages in some moderate-to-vigorous PA but is inactive for the remainder of the time. However, acute PA has been shown to have positive effects on executive function [76-80], whereas taking the mean PA might overlook the association between PA and state impulsivity. Because the time intervals differed considerably in length, the mean acceleration was the most straightforward operationalization of PA in this study. Future EMA studies should consider implementing a sampling approach that yields time intervals of similar length (see *Discussion* section) to allow the operationalization of PA as minutes engaged in different PA intensities (eg, minutes of moderate-to-vigorous PA). In addition, the differentiation between exercise and nonexercise PA could provide further insights, as found for the effect of PA on mood [81]. A further explanation for the positive association between PA and the probability to be impulsive could be the nature of MIS items. For instance, being impatient could manifest as walking up and down or twiddling with something. Spending more money than intended might be more likely to occur when being out and about (eg, walking around the city). This further highlights the importance of differentiating between different PA intensities and between exercise and nonexercise PA in future studies.

### Strengths and Limitations

This study is, to the best of our knowledge, the first to assess the relationship between macronutrient intake, PA, and state impulsivity in daily life using EMA. This kind of research is innovative and highly important to better understand fluctuations of ADHD symptomology in daily life and provides novel evidence of high temporal resolution and ecological validity, which is highly relevant to the growing field of Nutritional Psychiatry. However, the findings of this study should be interpreted in the light of some limitations. First, the assessment of impulsivity as well as food intake is based on self-reports. However, participants might be less likely to report foods and drinks and respond to prompts when being (more) impulsive, which might have caused some bias (ie, systematic noncompliance). Therefore, more objective assessments of dietary intake (eg, passive detection of eating events [82] and automatized photo-based dietary assessment) and impulsivity (eg, passive detection of impulsive behavior [83]) are desirable. However, although self-reports are generally prone to bias, particularly self-reports of food intake [84], assessing food intake in real time or near real time, as done in this study, minimizes recall biases compared with typically used retrospective dietary assessments (eg, FFQs). In addition, the smartphone app used for the dietary assessment in this study, the APPetite mobile app, was subject to a validation study, which showed that the app assessed food intake more accurately compared with widely used 24-hour recalls in healthy participants [51]. Second, this study did not allow the establishment of temporal sequences or causal relationships. Although EMA studies are essential to shed light on ecologically valid microtemporal associations between macronutrient intake, PA, and state impulsivity, complementary controlled studies are needed to gain insights into the directionality of the short-term effects of macronutrients and PA on state impulsivity. Third, the EMA period comprised only 3 days, which might not be sufficient to capture the complete spectrum of the associations of interest. However, given the high burden and time investment of the food recording, longer assessment periods would not have been feasible. Advanced dietary assessment methods for the naturalistic setting, such as wearable sensors passively detecting eating behavior and accurate automatized photo-based assessment of macronutrient composition, which require minimal user interaction, are required to allow prolonged EMA periods.

The strengths of this study were (1) the objective assessment of PA, (2) the application of sophisticated statistical models (ie, multilevel 2-part models), and (3) the inclusion of adults with and without ADHD. For instance, Abramovitch et al [43] used a self-report questionnaire to capture PA, although 2 systematic reviews indicated that indirect measures of PA (ie, self-reports) are unsatisfactory given that they differ substantially from direct, objective measures, such as accelerometers [85,86]. As impulsivity is widely understood as a characteristic that everyone shows some degree of—with clinical samples such as individuals with ADHD showing particularly high levels—it is especially interesting to study the association between macronutrient intake, PA, and state impulsivity in a clinical and control samples.

### Conclusions

This study is the first to apply EMA to assess short-term, microtemporal associations between macronutrient intake, PA, and state impulsivity in everyday life of adults with and without ADHD. Although EMA research in the context of Nutritional Psychiatry is still in its infancy, it is undeniably a highly promising and innovative approach to gain insights into microtemporal dynamics of psychiatric symptomology and lifestyle behaviors in daily life. This study provides and discusses important methodological considerations that can help advance the field and contribute to the optimization and tailoring of future EMA protocols. In addition, the findings from EMA studies can help build the foundation for the development of just-in-time adaptive interventions. This type of intervention represents a key element of digital psychiatry as it provides personalized support in daily life of patients right at the time it is needed most.

## Appendix

Multimedia Appendix 1 Open data.

Multimedia Appendix 2 Open R code.

Multimedia Appendix 3 Results of model 3 and 4 of the attention-deficit/hyperactivity disorder sample and control sample.

## Abbreviations

ADHD: attention-deficit/hyperactivity disorder
EMA: ecological momentary assessment
FFQ: food frequency questionnaire
MIS: Momentary Impulsivity Scale
OR: odds ratio
PA: physical activity
UPPS-P Impulsive Behavior Scale: Urgency-Premeditation-Perseverance-Sensation Seeking-Positive Urgency Impulsive Behavior Scale

## Data Availability

The data and R code that support the findings of this study are available in the Multimedia Appendices 1 and 2 of this paper.
