# Supplementary material for: Microtemporal Dynamics of Dietary Intake, Physical Activity, and Impulsivity in Adult Attention-Deficit/Hyperactivity Disorder: Ecological Momentary Assessment Study Within Nutritional Psychiatry
Source: JMIR Ment Health. 2023 Aug 17;10:e46550. doi: 10.2196/46550 (PMC10472180; doi:10.2196/46550)
Supplement: Multimedia Appendix 3 [file mental_v10i1e46550_app3.pdf]

Multimedia Appendix 3. Results of model 3 and 4 of the attention-deficit/hyperactivity disorder sample and control sample.

Table S1. Model estimates of the multilevel two-part model including an interaction between sugar and fat intake in the ADHD sample.

	Zero part				Continuous part			
	Estimate	SE	95% CI[a]		Estimate	SE	95% CI	
			LL[b]	UL[c]			LL	UL
				Model 3:				
**Fixed effects**								
intercept	−2.02	0.56	−3.17	−0.95	1.20	0.12	0.95	1.44
sugar intake	−0.02	0.11	−0.23	0.18	0.01	0.02	−0.03	0.05
sat. fat intake[d]	−0.10	0.34	−0.79	0.53	0.01	0.05	−0.09	0.11
protein intake	−0.10	0.14	−0.38	0.16	−0.02	0.02	−0.05	0.02
PA	−0.09	0.03	−0.15	−0.04	0.01	0.00	−0.00	0.01
gender	0.40	0.76	−1.07	1.90	−0.14	0.17	−0.48	0.20
age	−0.02	0.04	−0.09	0.05	0.00	0.01	−0.01	0.02
BMI	0.11	0.05	0.02	0.20	0.00	0.01	−0.02	0.02
trait-impulsivity	−0.11	0.07	−0.24	0.03	0.03	0.02	0.00	0.06
sugar intake*sat. fat intake	−0.07	0.15	−0.41	0.19	0.00	0.02	−0.03	0.03
**Random effects**								
SD(intercept)	1.85	0.33	1.31	2.60	0.44	0.06	0.33	0.58
SD(sugar intake)	0.12	0.09	0.00	0.35	0.03	0.02	0.00	0.07
SD(sat. fat intake)	0.32	0.25	0.01	0.95	0.04	0.03	0.00	0.11
SD(protein intake)	0.20	0.15	0.01	0.55	0.02	0.01	0.00	0.04
SD(PA)	0.06	0.04	0.00	0.15	0.01	0.01	0.00	0.03
SD(sugar intake*sat. fat intake)	0.25	0.20	0.01	0.74	0.02	0.02	0.00	0.06

[a]credible interval
[b]lower limit
[c]upper limit
[d]saturated fat intake

Table S2. Model estimates of the multilevel two-part model including an interaction between sugar and fat intake in the control sample.

	Zero part				Continuous part			
	Estimate	SE	95% CI[a]		Estimate	SE	95% CI	
			LL[b]	UL[c]			LL	UL
				Model 4:				
**Fixed effects**								
intercept	0.75	0.27	0.22	1.28	0.70	0.07	0.55	0.84
sugar intake	−0.05	0.04	−0.14	0.03	−0.01	0.01	−0.04	0.02
sat. fat intake[d]	−0.12	0.12	−0.35	0.11	0.01	0.04	−0.07	0.08
protein intake	−0.02	0.05	−0.12	0.08	−0.02	0.02	−0.05	0.02
PA	−0.03	0.01	−0.05	−0.01	0.01	0.00	0.00	0.02
gender	−0.51	0.32	−1.15	0.12	0.16	0.09	−0.01	0.33
age	−0.02	0.02	−0.05	0.02	−0.00	0.00	−0.01	0.01
BMI	0.03	0.04	−0.04	0.11	0.00	0.01	−0.02	0.02
trait-impulsivity	−0.20	0.04	−0.27	−0.13	0.05	0.01	0.03	0.07
sugar intake*sat. fat intake	0.09	0.05	−0.00	0.18	−0.01	0.02	−0.05	0.02
**Random effects**								
SD(intercept)	1.43	0.12	1.21	1.69	0.33	0.03	0.27	0.39
SD(sugar intake)	0.06	0.05	0.00	0.17	0.03	0.02	0.00	0.06
SD(sat. fat intake)	0.14	0.11	0.01	0.39	0.05	0.04	0.00	0.14
SD(protein intake)	0.06	0.04	0.00	0.16	0.02	0.02	0.00	0.06
SD(PA)	0.07	0.02	0.04	0.10	0.01	0.01	0.00	0.02
SD(sugar intake*sat. fat intake)	0.07	0.06	0.00	0.22	0.02	0.02	0.00	0.07

[a]credible interval
[b]lower limit
[c]upper limit
[d]saturated fat intake

Table S3. Model estimates of the multilevel two-part model including an interaction between sugar or fat intake (or both) and PA in the ADHD sample.

	Zero part				Continuous part			
	Estimate	SE	95% CI[a]		Estimate	SE	95% CI	
			LL[b]	UL[c]			LL	UL
				Model 5:				
**Fixed effects**								
intercept	−2.15	0.61	−3.39	−1.01	1.20	0.12	0.96	1.44
sugar intake	−0.14	0.14	−0.42	0.12	0.01	0.02	−0.03	0.05
sat. fat intake[d]	−0.01	0.41	−0.83	0.77	0.01	0.05	−0.09	0.12
protein intake	−0.12	0.16	−0.45	0.17	−0.02	0.02	−0.05	0.02
PA	−0.12	0.04	−0.20	−0.06	0.01	0.01	−0.00	0.02
gender	0.45	0.81	−1.11	2.07	−0.16	0.17	−0.49	0.17
age	−0.02	0.04	−0.09	0.05	0.00	0.01	−0.01	0.02
BMI	0.11	0.05	0.02	0.22	0.00	0.01	−0.02	0.02
trait-impulsivity	−0.11	0.07	−0.26	0.04	0.03	0.02	0.00	0.06
sugar intake*sat. fat intake	−0.20	0.21	−0.68	0.15	0.00	0.02	−0.03	0.04
sugar intake*PA	−0.03	0.03	−0.09	0.02	0.00	0.00	−0.01	0.01
sat. fat intake*PA	−0.01	0.06	−0.13	0.10	−0.00	0.01	−0.02	0.01
sugar intake*sat. fat intake*PA	0.02	0.05	−0.08	0.12	0.00	0.00	−0.01	0.01
**Random effects**								
SD(intercept)	1.98	0.36	1.39	2.78	0.43	0.06	0.33	0.57
SD(sugar intake)	0.14	0.11	0.01	0.41	0.03	0.02	0.00	0.08
SD(sat. fat intake)	0.49	0.36	0.02	1.33	0.04	0.03	0.00	0.13
SD(protein intake)	0.23	0.18	0.01	0.66	0.02	0.01	0.00	0.05
SD(PA)	0.06	0.04	0.00	0.15	0.01	0.01	0.00	0.03
SD(sugar intake*sat. fat intake)	0.38	0.28	0.02	1.07	0.02	0.02	0.00	0.06
SD(sugar intake*PA)	0.04	0.03	0.00	0.11	0.00	0.00	0.00	0.01
SD(sat. fat intake*PA)	0.08	0.07	0.00	0.24	0.02	0.01	0.00	0.04
SD(sugar intake*sat. fat intake*PA)	0.11	0.06	0.02	0.25	0.01	0.00	0.00	0.02

[a]credible interval
[b]lower limit
[c]upper limit
[d]saturated fat intake

Table S4. Model estimates of the multilevel two-part model including an interaction between sugar or fat intake (or both) and PA in the control sample.

	Zero part				Continuous part			
	Estimate	SE	95% CI[a]		Estimate	SE	95% CI	
			LL[b]	UL[c]			LL	UL
				Model 6:				
**Fixed effects**								
intercept	0.76	0.27	0.24	1.29	0.70	0.07	0.56	0.84
sugar intake	−0.05	0.05	−0.15	0.04	−0.01	0.02	−0.04	0.02
sat. fat intake[d]	−0.11	0.12	−0.35	0.14	0.02	0.04	−0.06	0.09
protein intake	−0.02	0.05	−0.12	0.08	−0.02	0.02	−0.05	0.01
PA	−0.03	0.01	−0.06	−0.01	0.01	0.00	0.00	0.02
gender	−0.52	0.33	−1.16	0.11	0.16	0.09	−0.01	0.33
age	−0.02	0.02	−0.05	0.02	−0.00	0.00	−0.01	0.01
BMI	0.03	0.04	−0.04	0.11	0.00	0.01	−0.02	0.02
trait-impulsivity	−0.20	0.04	−0.27	−0.12	0.05	0.01	0.03	0.07
sugar intake*sat. fat intake	0.09	0.05	−0.01	0.19	−0.01	0.02	−0.05	0.02
sugar intake*PA	−0.00	0.01	−0.02	0.01	0.00	0.00	−0.01	0.01
sat. fat intake*PA	−0.02	0.02	−0.06	0.02	0.00	0.01	−0.01	0.01
sugar intake*sat. fat intake*PA	−0.01	0.01	−0.03	0.02	−0.01	0.00	−0.01	0.00
**Random effects**								
SD(intercept)	1.43	0.12	1.21	1.69	0.32	0.03	0.27	0.39
SD(sugar intake)	0.06	0.05	0.00	0.18	0.03	0.02	0.00	0.07
SD(sat. fat intake)	0.16	0.12	0.01	0.44	0.05	0.04	0.00	0.13
SD(protein intake)	0.06	0.04	0.00	0.17	0.02	0.02	0.00	0.06
SD(PA)	0.07	0.02	0.03	0.10	0.01	0.01	0.00	0.02
SD(sugar intake*sat. fat intake)	0.10	0.07	0.00	0.28	0.03	0.02	0.00	0.08
SD(sugar intake*PA)	0.01	0.01	0.00	0.03	0.00	0.00	0.00	0.01
SD(sat. fat intake*PA)	0.03	0.02	0.00	0.07	0.01	0.01	0.00	0.02
SD(sugar intake*sat. fat intake*PA)	0.02	0.02	0.00	0.06	0.00	0.00	0.00	0.01

[a]credible interval
[b]lower limit
[c]upper limit
[d]saturated fat intake
